# Functional characterization of the dimeric form of PDGF-derived fusion peptide fabricated based on theoretical arguments

**DOI:** 10.1038/s41598-024-51707-2

**Published:** 2024-01-10

**Authors:** Maryam Sadeghi-Ardebili, Sadegh Hasannia, Bahareh Dabirmanesh, Ramazan Ali Khavari-Nejad

**Affiliations:** 1 Department of Biology, Science and Research Branch, Islamic Azad University, PO BoX 14515-775, Tehran, Iran; 2 Department of Biochemistry, Faculty of Biological Sciences, Tarbiat Modares University, PO Box 14115-175, Tehran, Iran

**Keywords:** Biochemistry, Biotechnology, Molecular biology

## Abstract

A skin wound leads to the loss of skin integrity and the influx of pathogens into the tissue. Platelet-derived growth factors (PDGFs) are cytokines released from alpha granules during wound healing and interact with their cell surface receptors and activate signals involved in chemotaxis, growth, proliferation, and differentiation pathways. Due to the low stability of growth factors (GFs), a new peptide-derived PDGF-BB was designed, expressed in the Shuffle strain of *E*. *coli*, and purified by Ni-NTA agarose affinity column chromatography. The effect of fusion peptide was then evaluated on L929 fibroblast cells and animal models with skin lesions. In vitro, studies showed that the peptide led to an increase in the migration of fibroblast cells in the scratch assay. Its positive effect on wound healing was also observed in the skin-injured rats after 3, 7, and 12 days. A significant rise in neutrophils and granular tissue formation, re-epithelialization, angiogenesis, and collagen formation was exhibited on the third day of treatment when compared to the control group. The results showed that, despite reducing PDGF size, the fusion peptide was able to maintain at least some of the known functions attributed to full-length PDGF and showed positive results in wound healing.

## Introduction

Wound healing is a natural biological process in the human body that is archived through four precise and highly regulated steps: homeostasis, inflammation, proliferation, and regeneration^1^. Many types of cells, cytokines, growth factors, and extracellular matrix (ECM) are involved in the healing process^2^.

Human Platelet-Derived Growth Factors (PDGFs) act as potent mitogens and chemical adsorbents for fibroblasts and mesenchymal cells and play a crucial role in many processes such as cellular proliferation, differentiation, wound healing, embryonic development, inflammation, survival, and migration^3^. PDGFs are dimer glycoproteins with four isoforms named PDGF-A, PDGF-B, PDGF-C, and PDGF-D^4^. Among these compounds, PDGF-BB homodimer (molecular weight: 14 kDa) is the most abundant in the body^5^. PDGFs are mainly secreted by damaged platelets, fibroblasts, and vascular endothelial cells^6^. Their production and secretion increase during wound formation, guiding neutrophils, macrophages, fibroblasts, and smooth muscle cells to the wound site leading to the initiation of the inflammatory response. It also contributes to cell differentiation and maturation/morphology by reorganizing actin filaments^7^.

In recent years, scientists have focused their efforts on the effect of compounds containing growth factors on the wound-healing process. The results of their studies mainly indicate the positive impact of growth factors on the quality and speed of wound healing^8^. Becaplermin topical ointment (Regranex^®^) which was approved by the US Food and Drug Administration in 1997, is used to treat chronic diabetic foot ulcers in diabetic patients and contains recombinant human PDGF-BB^9^. Other recombinant drugs produced to treat diabetic foot ulcers include Heberprot-P (epidermal growth factor)^10^, Repifermin (fibroblast growth factor)^11^, and Telbermin (vascular endothelial growth factor-A)^12^.

Based on previous studies, it seems that PDGF could improve the process of wound healing and vascular regeneration. With the increasing number of patients with acute and chronic wounds and the use of PDGF in the ossification of dental surgeries, the recombinant production of this growth factor is considered to accelerate. However, due to the high cost and low half-life of PDGF, several groups have used different approaches to decrease its effective dose and increase its stability. Recombinant peptides derived from the PDGF-B chain have been designed to act as PDGF agonists or antagonists. Engstrom et al. introduced an antagonist 13-amino acid peptide (ANFLVWEIVRKKP) from residues 116 to 121 and 157 to 163 of the PDGF-B chain^13^. Zamora et al. also reported a PDGF agonist peptide by linking residues 153–162 on the PDGF-B chain (VRKIEIVRKK) to a heparin-binding sequence (RKRKLERIAR)^14^. In addition, Deptuła and colleagues designed three peptides based on the L1 and L3 chains of PDGF that showed pro-proliferative effects on human skin cells, high immunological safety, low cytotoxicity, and accelerated wound healing in the mouse model^2^.

Peptides are cost-effective small molecules with high regenerative power that have been used for targeting specific regions of target proteins^15^. These biocompatible compounds are easily synthesized and have controllable properties, including size and functional groups that make them perfect molecules with potentially significant impact on healing^2^.

For the above-mentioned reasons, in the current study, a PDGF-BB-derived fusion peptide was designed. This fusion peptide contained parts of PDGF-BB loop III, β3, and β4 sheets as a receptor binding site and hydroxy apatite affinity peptide as a spacer. The structure of the designed fusion peptide was first predicted by molecular dynamic simulation and docking analysis. Then the gene was optimized, synthesized, and cloned into pET21a(+). The recombinant construct was expressed, and purified and its effect was investigated on the viability and migration of fibroblast cell line L929 using a wound healing assay. Positive results from the previous stage encouraged us to examine the in vivo functionality of this fusion peptide using a rat skin wound model. Results revealed that this construct could be a potential candidate for inducing wound closure.

## Materials and methods

### Bacterial strains and culture media

Here, SHuffle^®^ *E. coli* cell (Novagen, Germany) was used as a host bacteria. The Ni-NTA agarose chromatography column and IPTG were purchased from Biobasic (Canada). Peptone, agar, and yeast extract were obtained from Biobasic (Canada). Anti-PDGF-BB antibody and HRP secondary antibody were purchased from Santa Cruz Biotechnology, (Santa Cruz, CA).

### Modeling

The MODELLER software v 10.2 was used to construct the PDGF-BB-derived structure. The structure was modeled based on the sequence and the crystallographic structure of the platelet-derived growth factor receptor with PDB ID: 3MJG (the structure of a platelet derived growth factor receptor complex) as the modeling template.

### MD simulation

AMBER software was used to perform molecular dynamics simulations. Initially, the steepest descent algorithm was used to minimize energy. The minimized structure was then subjected to temperature equilibration for 100 ps (278 K) (NVT stage). Pressure balancing also continued for 755 ps (NPT stage). Finally, the simulation of molecular dynamics continued for 30 ns.

### Docking

After optimizing the protein structure using molecular dynamics simulations, the binding of the peptide to the receptor was investigated using the molecular docking method. ClusPro software v 2.0 was used for docking. For this purpose, the stabilized peptide fusion file was selected from the molecular dynamic stage and its binding to PDGFR was investigated.

### Expression and purification

The DNA sequence of the fusion peptide was synthesized and cloned into pET21-a(+) by GenScript company. Subsequently, the recombinant plasmid was transformed into *E*. *coli* SHuffle^®^ strain and grown on an LB-agar plate containing 100 µg ml^−1^ of ampicillin. A colony was inoculated into LB broth containing appropriate antibiotic overnight at 37 °C.

Culture media of 2xyT (16 g tryptone, 10 g yeast extract, and 5 g NaCl, up to 1000 ml) was inoculated by pre-culture inoculum (1%) and incubated at 23 °C with shaking (180 rpm). At OD_600_ of 0.5, the recombinant fusion peptide was induced with 0.5 mM IPTG for 6 h.

Finally, the culture was harvested by centrifugation at 4000×*g* for 5 min. The cell pellet was resuspended in 50 mM NaH_2_PO_4_ pH 7.2, 300 mM NaCl, and 10 mM imidazole lysis buffer and sonicated on ice for 30 min. The lysate supernatant was loaded onto a Ni-NTA agarose chromatography column. Impurities were removed with an initial buffer containing 25 mM imidazole. The recombinant fusion peptide was eluted by 250 mM imidazole. Purification of the recombinant protein was confirmed by SDS-PAGE 12.5% and Western blotting. The purified protein was then dialyzed against PBS buffer (two times 4 h) at 4 °C. After dialysis, the protein was concentrated using polyethylene glycol 6000 (Neutron Company).

Ellman’s reagent, 5,5′-dithiol-base-(2-nitrobenzoic acid) (DTNB), reacts with free thiol groups to produce a yellow dye. Therefore, it measures the presence of thiol groups on a protein^16^. The reagent was prepared with a concentration of 4 mg ml^−1^ DTNB in 100 mM sodium phosphate pH 8.0 and 1 mM EDTA. A cysteine stock solution was prepared with a final concentration of 2.05 mM in 100 mM sodium phosphate buffer and 1 mM EDTA. Different cysteine concentrations were mixed with Ellman’s reagent in the presence of 100 mM sodium phosphate buffer and 1 mM EDTA at room temperature for 15 min. Absorption at 412 nm was measured against a blank (100 mM sodium phosphate buffer (pH 8.0), 1 mM EDTA, and DTNB reagent).

### Cell viability

Cell viability was assessed using MTT (3-(4,5-dimethylthiazol-2-yl)-2,5-diphenyltetrazolium bromide) assay. Mouse fibroblast cell line L929 was purchased from the Pasteur Institute of Iran. Cells were cultured in Dulbecco’s modified Eagle’s medium (DMEM) (Gaithersburg, USA) containing l-glutamine (Gaithersburg, USA), 10% FBS (Gaithersburg, USA) and 1 × Penicillin/Streptomycin (Gaithersburg, USA) at 37 °C with 5% CO_2_ in a humidified atmosphere. In all experiments, cells were seeded with a density of 2 × 10^4^ cells per well. After 24 h, the cell medium was replaced by a new culture medium containing 5% FBS and different concentrations of the fusion peptide (0.18, 0.36, 0.72, 1.45, and 2.9 µg ml^−1^). After 24 h, 10 μl MTT was added to each well with a final concentration of 0.05 mg ml^−1^ and incubated for 3 h at 37 °C. Then, the culture medium was removed, and the formed Formazan crystals were dissolved in 150 μl DMSO (5%). The light absorption of each well was read at 540 nm using an ELISA plate reader.

### Scratch assay

The L929 cells were seeded at 5 × 10^4^ cells ml^−1^ in a 24-well plate. When the L929 cells reached a 70–80% confluence, wounds were created by a sterile pipette. Cell debris was cleared by rinsing with PBS. Then, they were treated with the fusion peptide at a concentration of 3 µg ml^−1^ for 6, 24, 48, and 72 h. The cells were then stained with Giemsa dye and the migration distance was measured by ImageJ software.

### Animal modeling-gross examination of wound

Nine Wistar Albino Rats (n = 9), were divided into 3 groups each containing three rats and a weight range of 200 + 50 g. The animals were sourced from the National Animal Breeding Center (from Razi Vaccine and Serum Research Institute, Karaj, IRAN) to investigate the effect of the fusion peptide on the wound healing process. They were maintained at an animal facility with controlled laboratory conditions; the temperature was 22 ± 2 °C, a 12-h light–dark cycle, and a relative humidity range of 40–60%. During this time, adequate water and standard food were provided to them. Anesthesia was induced via intramuscular injection of a 10% ketamine and Xylazine mixture in a 3:1 ratio.

Following anesthesia, the hair on their backs was completely shaved, and the area was disinfected with betadine. Using a biopsy skin punch, two full-thickness wounds with an outer diameter of approximately 8 mm were created in the dermis and hypodermis according to surgical guidelines on the back of each rat (Left, and Right)^17^.

Post-injury, the rats were placed in individual cages, and lidocaine spray was applied to alleviate pain. The wounds were treated with either the fusion peptide or PBS (used as a negative control). The first day after wounding was designated as day zero. The groups received local treatment with 4.5 µg ml^−1^ of the fusion peptide twice daily. The wound area was assessed using a caliper on days 3, 7, and 12, and photographs were taken on these days to visually evaluate wound quality and appearance. No adverse effects related to injury or anesthetic drugs were observed during the study. The research protocol was approved by the Islamic Azad University–Tehran Science and Research Branch Ethics Committee (ID: IR.IAU.SRB.REC.1398.183) and conducted in accordance with the Guideline for the Care and Use of laboratory Animals in Iran^18^.

All experiments of the current study were also reported following ARRIVE guidelines^19^.

### Animal modeling-histology

Samples of total skin were taken from each group on days 3, 7, and 12 post-treatment (n = 3 each day) Samples were fixed and stored in 10% formalin solution for 24 h and washed with saline solution, then dehydrated by different percentages of alcohol. Xylol was used to remove the alcohol. Tissue samples were sliced to a thickness of 10–15 µm. For histological examinations, in addition to direct tissue photography, tissue staining with Hematoxylin and Eosin (H&E) and Mason trichrome were performed. Stained tissue sections were investigated under a microscope, magnifying × 20, × 100, and × 200. Percentage of wound contraction are was estimated following Eq. (1):

1 $$\% \;{\text{of wound contraction}} = \left[ {\left( {{\text{W}}1 - {\text{W}}0} \right)/{\text{W}}1} \right] \times 100$$

where W1 and W0 represent the initial wound size and wound size of the specific day respectively.

### Statistical analysis

In this study, the values presented in the graphs are mean ± SD. Student *t*-test statistical analysis was performed to compare the two groups and one-way or two-way way ANOVA was used for analyzing more than two groups with turkey tests for multiple samples and unpaired *t*-tests for two groups. A p-value less than 0.05 is statistically significant. The p-values less than 0.05, 0.01, and 0.001 are shown with one, two, and three asterisks, respectively.

## Results

**Figure 1:**
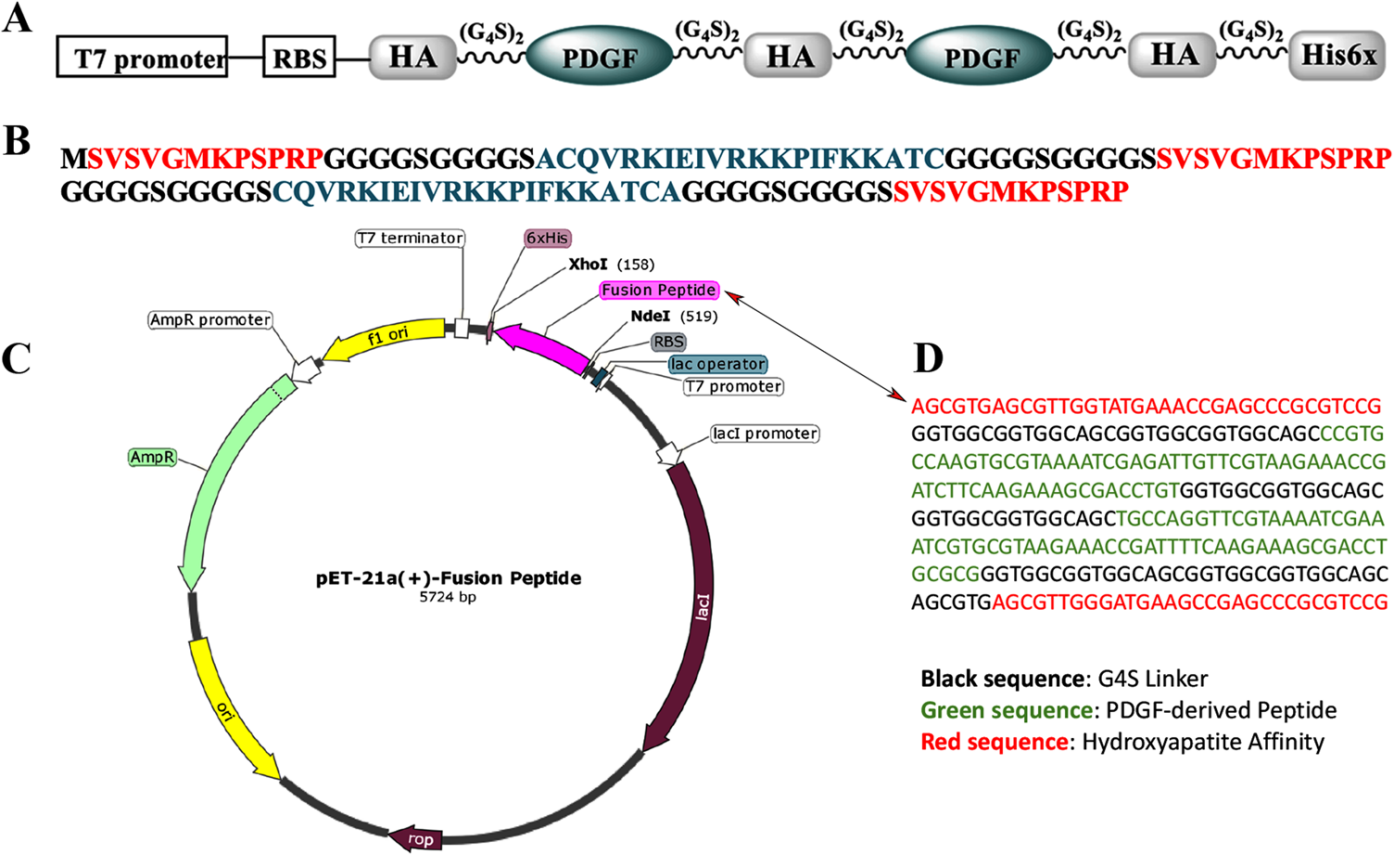
Diagram of the the DNA sequence of fusion peptide into pET21-a (**A**) representation of the designed construct, (**B**) Protein sequence of fusion peptide (**C**) diagram of the expression vector (**D**) DNA sequence of fusion peptide.

In this study, we designed a peptide containing two repeats of the DNA sequence encoding from Gln71 (β3), Loop III, and Tre88 (β4) (18 amino acids) of the PDGF-BB growth factor and two amino acids (Ala and Cys) in N-terminal and Cys added in C-terminal) (ACQVRKIEIVRKKPIFKKATC = 21 amino acids) that are linked by the (G4S)2 linker sequence and ended by the two hydroxyapatite affinity (SVSVGMKPSPRP) peptides in N and C-terminals, one in middle as a spacer and His6x sequences in C-terminal (Fig. 1). The desired fusion peptide was designed to trigger the PDGF signaling pathway by using only the binding domain. In this research, this fusion peptide was designed and made with several goals in mind. The first novelty is that, instead of a single peptide as has been reported before many times, we constructed a dimeric form of PDGF-BB derived peptides the same as native dimeric PDGF for the second item, the design of fusion peptide, we fused the two distinct PDGF derived peptides with the hydroxyapatite (HA) affinity peptide as a spacer and the same two peptides as tags in the amino and carboxyl-terminals of fusion form, that could be carried by HA scaffold component. This HA spacer and two HA tags can increase the molecular size of fusion peptides, facilitating their expression and decreasing washout from The HA scaffold.

The reduced size of the growth factor resulted in its increased stability. To increase its stability, four cysteine amino acids were considered to form two disulfide bonds in the amine and carboxyl-terminal of the selected sequence from the N-terminal of β3 and the C-terminal of β4.

### Modeling and MD simulation

**Figure 2:**
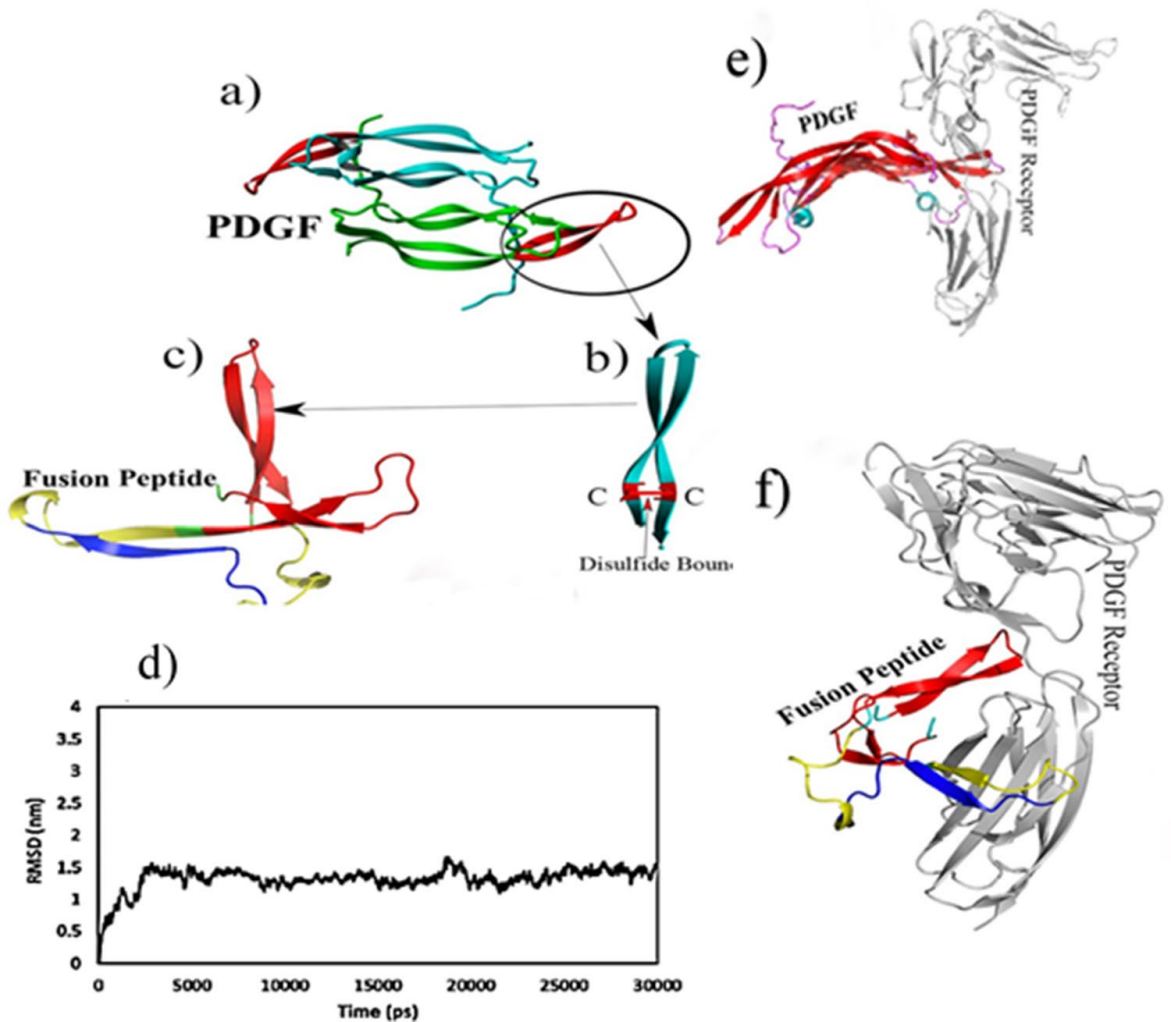
Molecular dynamic simulation of PDGF-BB derived peptide. (**a**) Ribbon model of the PDGF-B dimer from PDB ID 1PDG, (**b**) Section of β3-loop III-β4 with disulfide bond (**c**) Optimized model of the fusion peptide by AMBER, (**d**) RMSD curve, (**e**) Complex model of interaction between PDGF (**f**) and the fusion peptide with the receptor.

The initial structural studies of the fusion peptide were evaluated by computational analysis. The desired gene constructs were then ordered for synthesis. The fusion peptide contained two repeats of 18 amino acids of β3-loop III-β4 (Gln71–Thr88), three repeats of hydroxyapatite sequences, and a His6x tag in the C-terminal. The PDGF-derived sequence was modeled based on the X-ray crystallographic structure of the PDGF receptor complex with PDB code: 3MJG. The best model was selected based on the position of amino acids in the second and third structures and the most stable state of rotational angles. No specific model was considered for the G_4_S linker, hydroxyapatite, and His_6x_ affinity tags, so the molecular dynamics simulation method was used to optimize the structure of these regions. The best parameter to evaluate the structural stability of the protein is the root-mean-square deviation (RMSD) factor over time simulation. This factor represents the average displacement of alpha carbon atoms of amino acids within the peptide. The results of the molecular dynamics showed that it reached the value of 1.5 nm in 30 ns and remained at the same value until the end of the simulation time, which indicated that the structure was stable during the simulation time. The three-dimensional structure of the created model at the end of the simulation time and stable phase is shown in Fig. 2. Based on the docking results, the binding site of the selected fusion peptide overlapped with the PDGF receptor binding site and bound to the receptor at the same regions.

### Overexpression and purification

**Figure 3:**
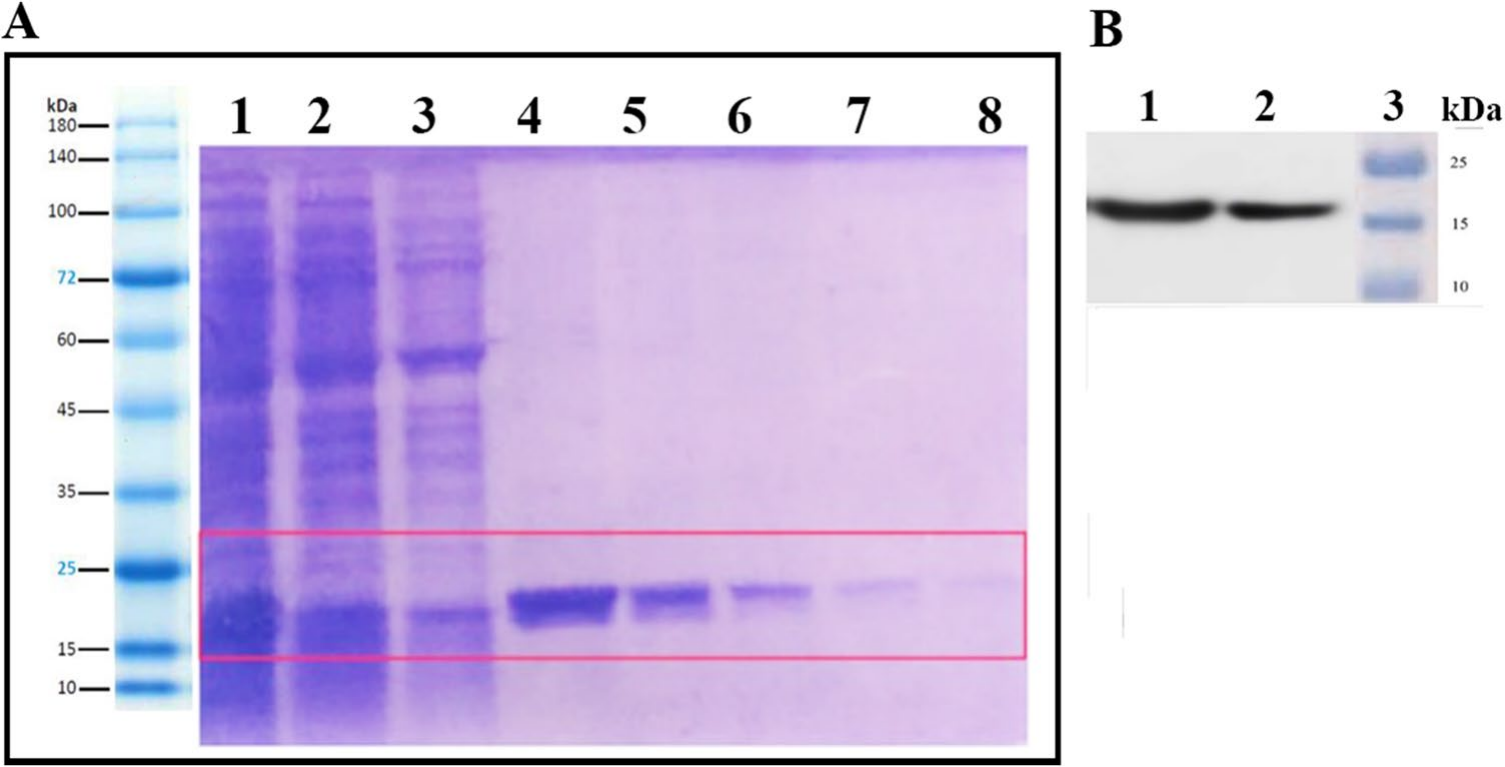
(**A**) SDS-PAGE analysis of expression and purification of the fusion peptide by Ni-NTA agarose lane 1: supernatant of *E. coli* lysate, lanes 2 and 3: washing buffer with 25 mM imidazole, lanes 4–8: elution buffer using 250 mM imidazole, (**B**) western blot analysis, lane 1: soluble protein, lane 2: purified protein, lane 3: protein marker. For more details, see Fig. 3, Fig. 3-S1, Fig. 3-S2 and Fig. 3-S3 in the supplementary file.

The recombinant vector was transformed into a SHuffle^®^ *E. coli* strain. The fusion peptide overexpression was investigated in different conditions such as culture medium, IPTG concentrations, induction temperature, and time. 2xYT medium was selected as the best-overexpressed medium that showed the highest overexpression when induced with 0.5 mM IPTG at 23 °C for 6 h. The recombinant fusion peptide was then purified by a Ni-NTA agarose affinity column and confirmed by SDS-PAGE and the western blotting analysis using a specific polyclonal antibody for PDGF-BB (Fig. 3). The SDS-PAGE and western blotting analysis of the soluble fraction showed a 17 kDa band. The purified fusion peptide was finally concentrated to 0.33 mg ml^−1^ using PEG 6000. Due to the presence of two disulfide bonds in the fusion peptide, the Ellman method was used to evaluate their formation in the structure. The absence of yellow color and no adsorption at 412 nm indicated the involvement of the thiol groups in the disulfide bond.

### Cell toxicity

**Figure 4:**
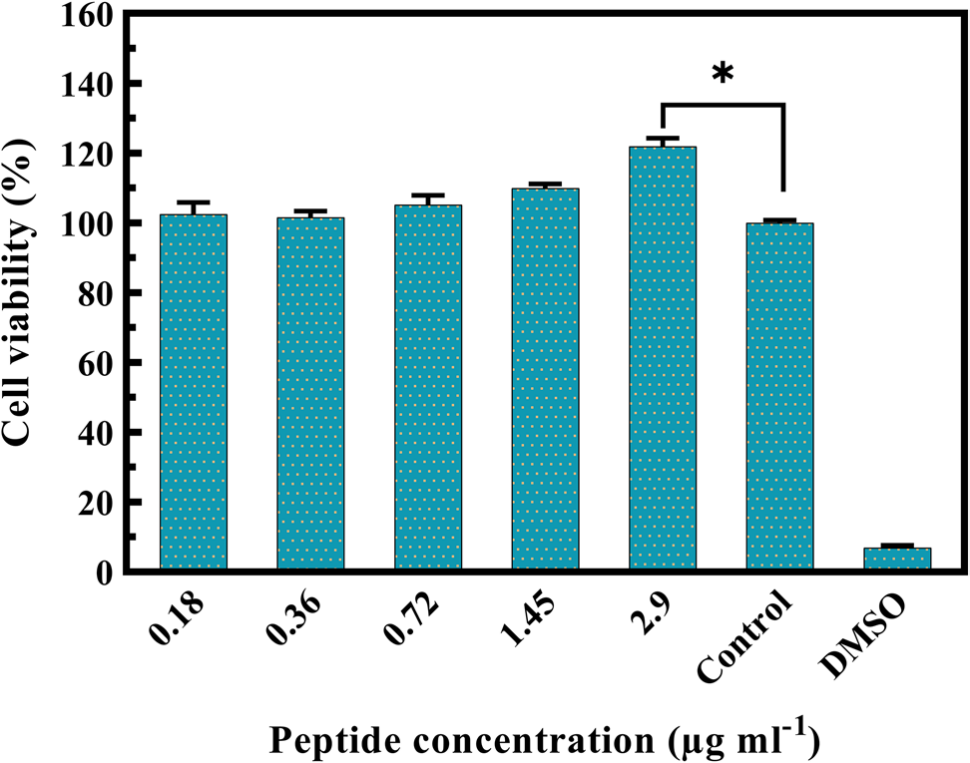
Cytotoxicity of the fusion protein. The cell viabilities (mean ± SEM) of L929 fibroblast cells were determined by the MTT assay in various peptide concentrations at 24 h. The mean difference between the data and the control (+ FBS) group of the same group was considered significant (*p < 0.05).

The L929 fibroblast cells were seeded in 96-well plates and grew to 50% confluence. They were then treated with different concentrations of the fusion peptide for 24 h. An increase in viability was observed up to 2.9 µg ml^−1^ when compared to the control group (Fig. 4).

**Figure 5:**
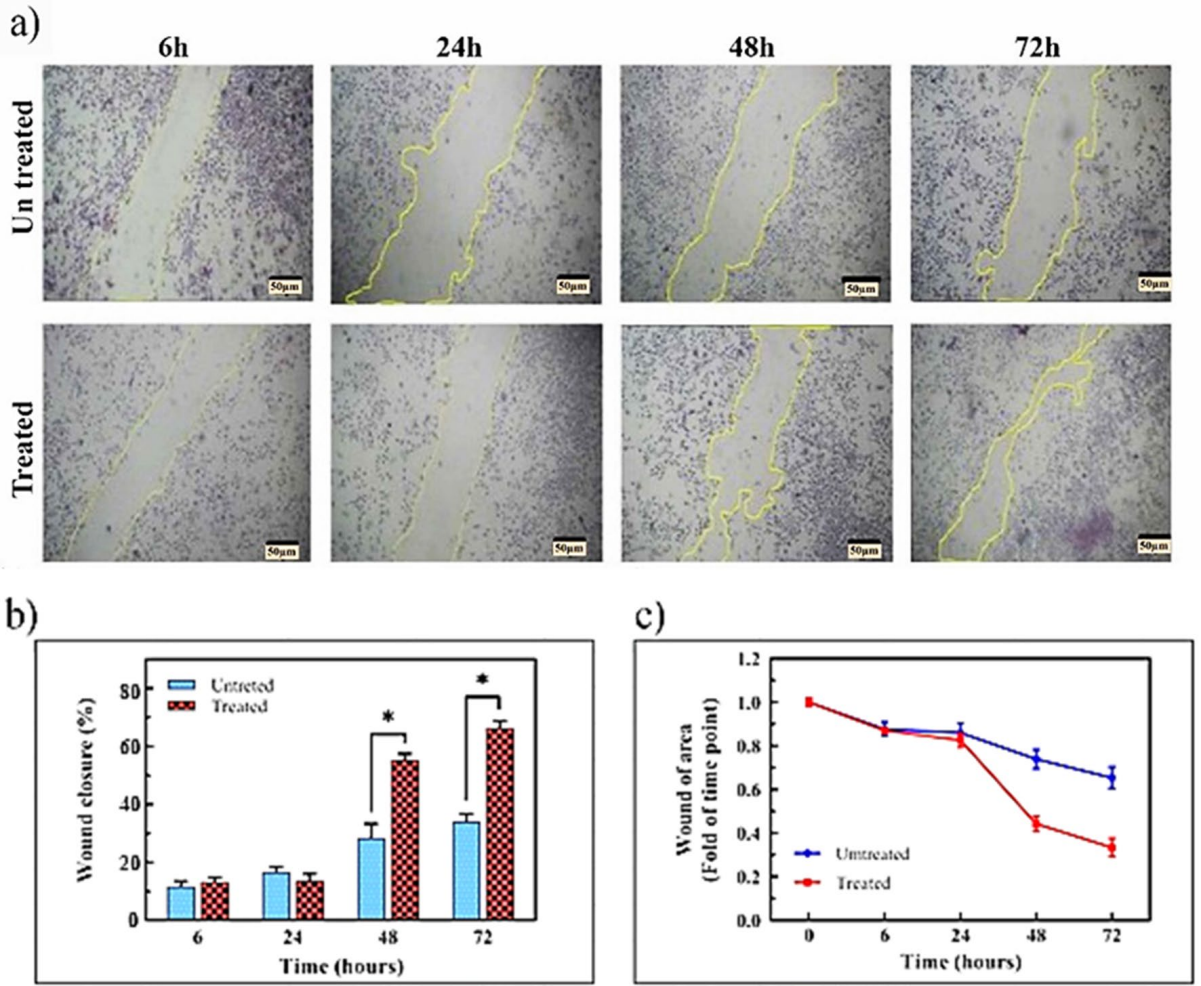
Evaluation of the effect of 3 µg ml^−1^ fusion peptide on the L929 fibroblast cell migration at 6, 24, 48, and 72 h. During the treatment time, the cell migration rate increased significantly (**a**), which is inversely correlated with the wound level (**b**), while it increases wound closure (**c**). The mean difference between the data and the control group of the same group was considered significant (*p < 0.05). Untreated: Received PBS and treated samples with 4.5 µg ml^−1^ peptide. Percentage value of mean ± SEM of each group.

Cell migration is an essential factor in wound healing. Therefore, L929 cell line migration was evaluated for 6, 24, 48, and 72 h in the absence and presence of the fusion peptide (3 µg ml^−1^). The scratch assay indicated an increase in cell movement after 6 and 24 h treatment, while the cell migration improved significantly after 48 and 72 h compared to the untreated cells (Fig. 5). Values of mean ± SEM of each group. Wound closure percentages of 6, 24, 48 and 72 h post treatments were reported as follows respectively. 13.23 ± 1.54 in treated (treated with peptide) groups and 11.67 ± 1.8 in untreated (PBS received) groups after 6 h, 16.70 ± 1.68 in treated (treated with peptide) groups, and 13.57 ± 2.44 in untreated (PBS received) groups after 24 h, 55.33 ± 2.14 in treated (treated with peptide) groups and 28.50 ± 4.82 in untreated (PBS received) groups after 48 h, 66.37 ± 2.55 in treated (treated with peptide) groups and 34.13 ± 2.68 in untreated (PBS received) groups after 72 h.

### Animal modeling-histology

The wound healing process is a very complex local process in which cells and other blood compounds are effective. The simulation of these interactions has severely limited the use of laboratory models, however, wound healing has been extensively studied in animal models. In particular, the rat has a special place in vivo studies due to its low cost and the possibility of rapid study with large numbers for statistical validation. Among the limitations of the study in these species, we can mention its small size and the primary mechanism of wound healing that is not similar to humans^20^. On the other hand, due to ethical regulations for the use of human models with skin injuries is prohibited, Rats were used, in addition to preventing pregnancy and hormonal changes affecting the male rats selected.

**Figure 6:**
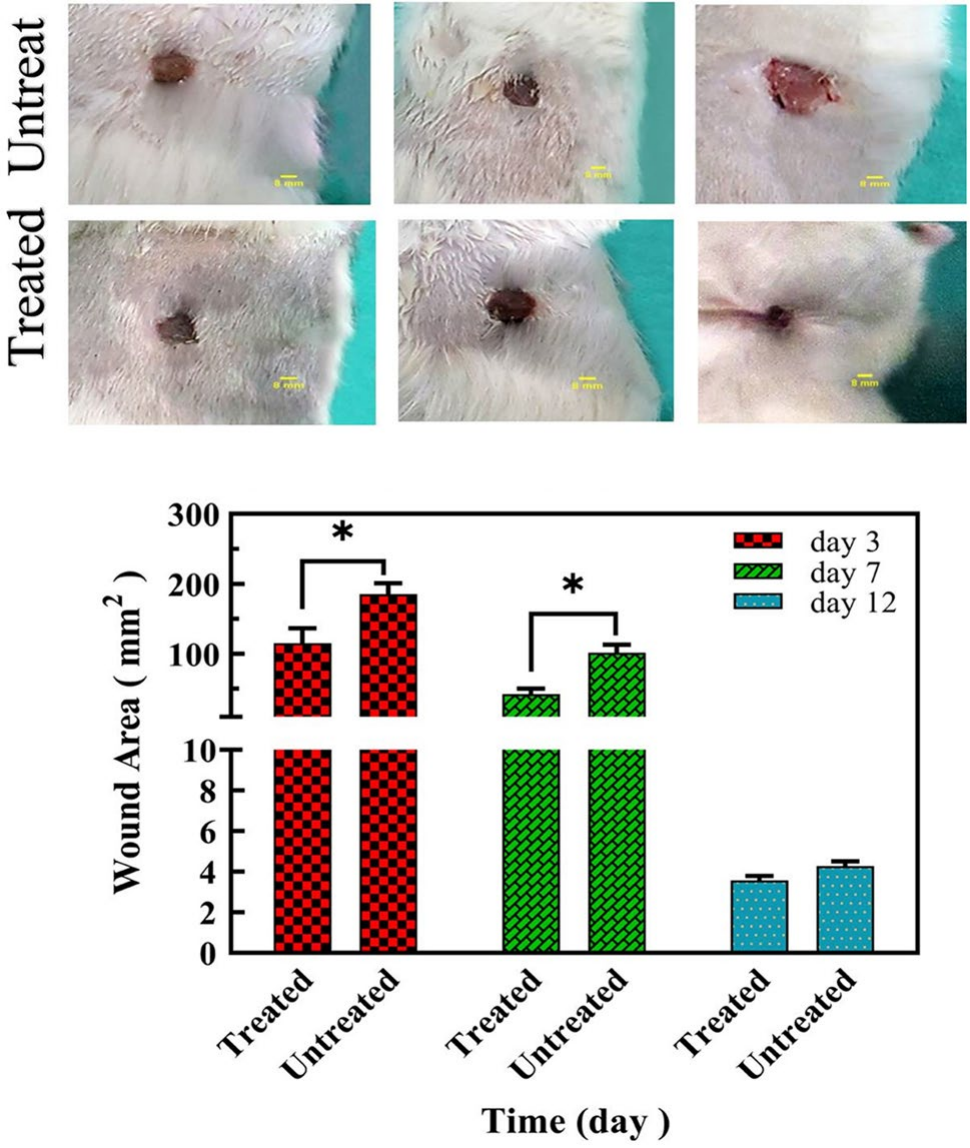
Evaluation of wound dimensions and wound healing process on the third, seventh, and twelfth days. (**a**) Over time, the wound healing rate increased in the treated group with 4.5 µg ml^−1^ fusion peptide, (**b**) diagram of wound measurement on the third, seventh, and twelfth days by caliper. A significant difference was observed between the wound dimensions in the treated group on the third and seventh days. The results are three repeats in each group on the third, seventh, and twelfth day (n = 3). Each rat has two full-thickness wounds on the back. Then, the right wounds received 4.5 µg ml^−1^ of the fusion peptide twice daily, and the left wounds were treated with PBS (as a negative control group). Untreated: received PBS and treated samples with 4.5 µg ml^−1^ peptide. Values of mean ± SD of each group. For more details, see Fig. 6, Fig. 6-a, Fig. 6-b in the supplementary file.

To investigate the therapeutic effects of the fusion peptide on wound healing, two 8 mm diameter wounds were made on the back of each rat using a scalpel (left and right). Then, the right wounds were treated twice daily with 4.5 µg ml^−1^ of the fusion peptide, and the left wounds were treated with PBS (as a negative control group). Imaging results were confirmed by the measurement of the external dimensions of the wound with a caliper on days 3, 7, and 12. The results indicated a significant effect on the quality and speed of healing in the treated groups compared to the negative control group. Over time, the wound healing rate increased in the treated group with 4.5 µg ml^−1^ fusion peptide. Starting on day 3, the wound size in the treatment group was significantly smaller than in the control group. On the seventh day, complete wound closure was observed in the fusion peptide-treated group. However, the wound in the control group was completely healed after twelve days (Fig. 6). Values of mean ± SD of each group. Wound area percentages on day 3, 7 and 12 post treatments were reported as follow respectively: 95.77 ± 24.42 in treated (treated with peptide) groups and 185.3 ± 28.19 in untreated (PBS received) groups on day 3, 42.91 ± 12.16 in treated (treated with peptide) groups and 101.5 ± 19.94 in untreated (PBS received)groups on day 7, 3.58 ± 0.38 in treated (treated with peptide) groups and 4.28 ± 0.41 in untreated (PBS received) groups on day 12.

The results of H&E staining exhibited a significant effect of the fusion peptide on the wound healing process in the treated group compared with the negative control group. On the third day of treatment, the wound is still in the early stages of healing (the inflammatory phase) and the number of neutrophils to defend against infectious agents in both treatment and control groups is increased. On the seventh and twelfth days, the neutrophil count decreases sharply, indicating wound healing and transition from the inflammatory to the regenerative phase. Wound granulation and epithelialization are complex processes that begin with collagen formation, small blood vessels, and capillaries, eventually forming connective tissue to fill the wound space. On the third day of treatment, the amount of granulation increased significantly compared to the control group and caused the formation of pink tissue in the wound. However, no significant difference was observed on the seventh day and especially on the twelfth day post-treatment. Based on H&E staining, on the first days of wound healing (third day), the epithelialization process did not occur in the control and treated groups. But on the seventh and twelfth days, the rate of re-epithelialization improved significantly, the wound bed was covered with keratinocytes and dermal layers were formed, at the same time, granular tissue in the bed was formed and keratinocyte migration increased.

**Figure 7:**
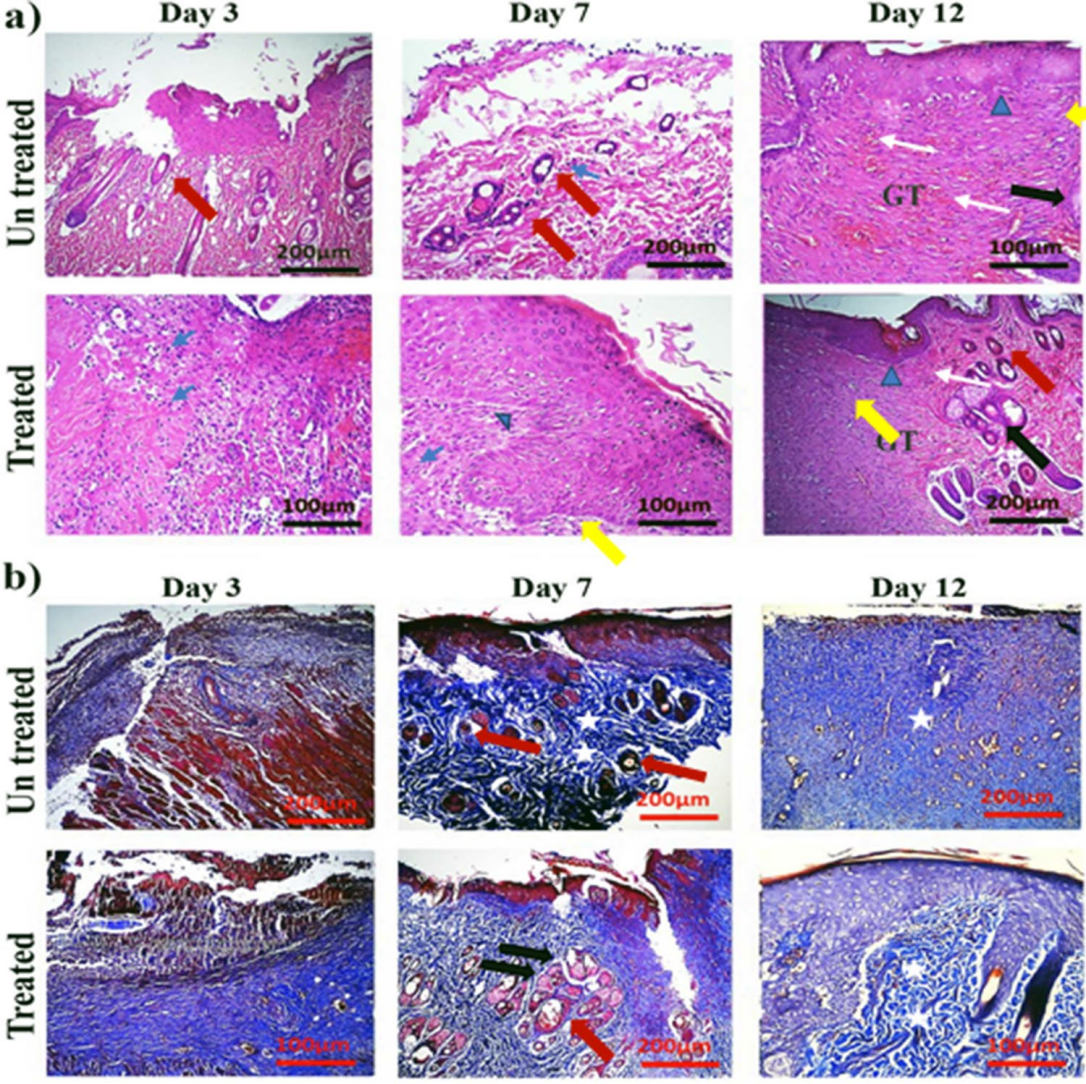
Histological evaluation of tissue changes in the control and treated group by hematoxylin–eosin (H&E) (**a**) and Mason's trichrome (**b**) staining methods at three time-points: days 3, 7, and 12. (n = 3 each day) Untreated: Received PBS and treated samples with 4.5 µg ml^−1^ peptide. Thick red arrow—hair follicle, thick black arrow-—sebaceous gland, thin blue arrow—neutrophil, blue head arrow—new epidermis, yellow arrow—dermis. Thin white arrow—blood vessels, white star—collagen, GT—granulation tissue.

**Figure 8:**
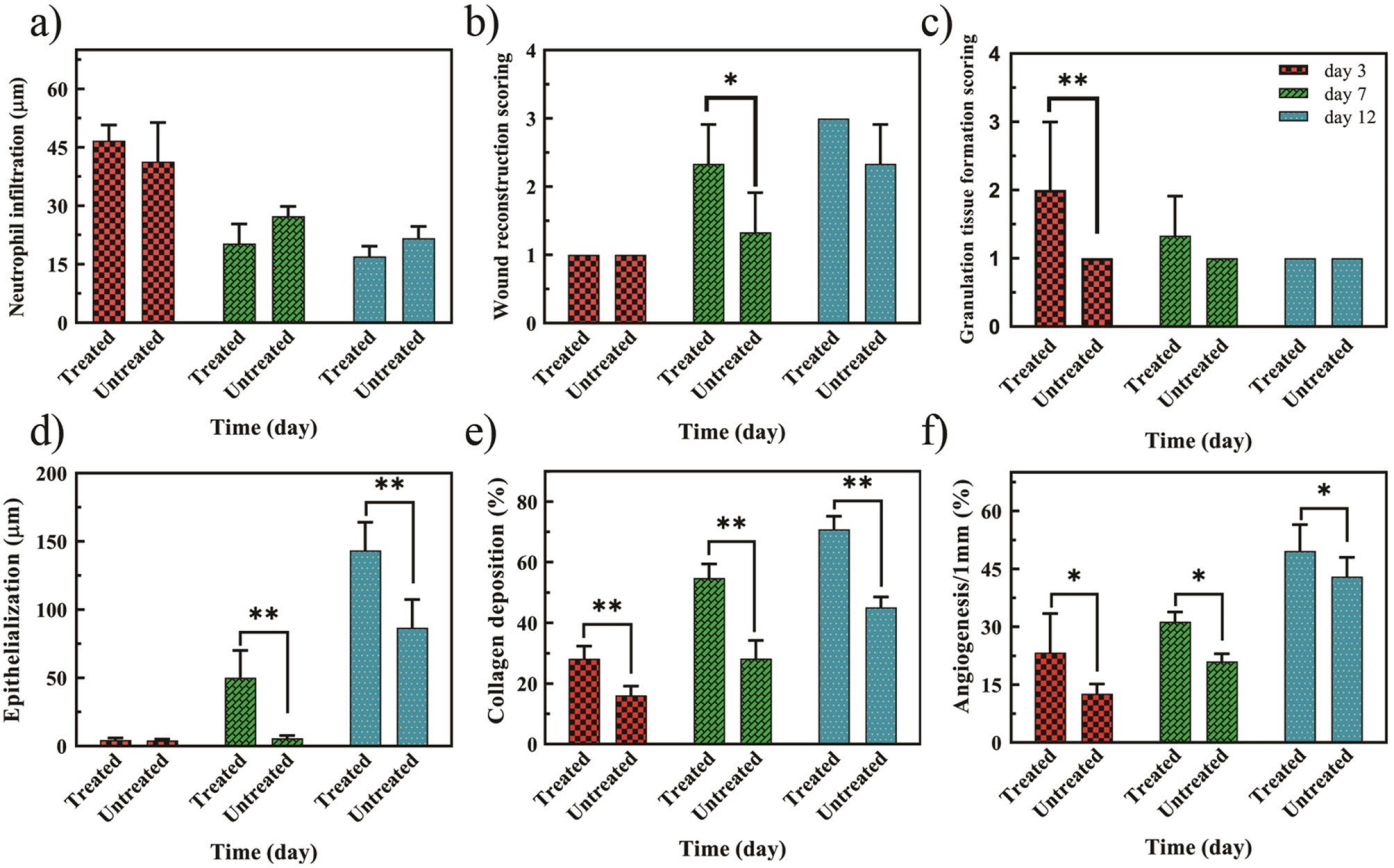
Histological analysis of the control and treated groups by H&E and Mason's trichrome. (**a**) Neutrophil infiltration, (**b**) wound healing, (**c**) granulation tissue formation, (**d**) epithelialization, (**e**) collagen level, (**f**) angiogenesis. The mean difference between the data and the control group of the same group was considered significant at (*p < 0.05 and **p < 0.01). Untreated: Received PBS and treated samples with 4.5 µg ml^−1^ peptide. Percentage value of mean ± SD of each group. Neutrophil infiltration score 0–3 (none, 0; 0–15% mild, 1; 15–30% moderate, 2; 30–45% severe, 3). Wound reconstruction score was between (1–3). 1 (below 25%) belongs to a poor healing, 2 (25–75%) belongs to medium and (up to 100%) belongs to a perfect healing. Further. The score value of new epithelization is estimated according to the literature. Scales (1–5) 0 = 0% healing, 1 = 1–25%, 2 = 26–50%, 3 = 51–75%, 4 = over 75% but not perfect and 100% assigns to 5. Finally, GT or granulation tissue was defined precisely from to 4 scores. 1 (1–25%) means thin GT, 2 (25–50%) means medium GT, 3 (50–75%) means thick GT, 4 (75–100%) means perfect thick GT similar to intact dermis. Collagen deposition scores: Score: 0, None; 1, scant; 2, moderate; 3, abundant 4. and Angiogenesis scores were described by a graded scale of 0–4 0 mean none and 4 means 100%.

Mason's trichrome staining method investigates the amount of collagen production and angiogenesis. The stained slides showed a significant increase in collagen production in the treated groups compared to the negative control. The collagen percentage was 35%, 55%, and 70% on days 3, 7, and 12 of treatment, respectively. The vascular formation increased in the fusion peptide treatment group and was higher on day 12 compared to days 3 and 7 (Figs. 7, 8). Untreated: Received PBS and treated samples with 4.5 µg ml^−1^ peptide. Percentage value of mean ± SD of each group. The neutrophil infiltration score was calculated according to a value scale of 0–3 (none, 0; mild, 1; moderate, 2; severe, 3). (none, 0; 0–15% mild, 1; 15–30% moderate, 2; 30–45% severe, 3). Wound reconstruction scores were between (1–3). 1 (below 25%) belongs to poor healing, 2 (25–75%) belongs to medium and (up to 100%) belongs to perfect healing. Further. The score value of new epithelization is estimated according to the literature. Scales (1–5) 0 = 0% healing, 1 = 1–25%, 2 = 26–50%, 3 = 51–75%, 4 = over 75% but not perfect and 100% assigns to 5. Finally, GT or granulation tissue was defined precisely from to 4 scores. 1 (1–25%) means thin GT, 2 (25–50%) means medium GT, 3 (50–75%) means thick GT, 4 (75–100%) means perfect thick GT similar to intact dermis. Collagen deposition scores: Score: 0, None; 1, scant; 2, moderate; 3, abundant, and angiogenesis scores were described by a graded scale of 0–4. Moreover, all detail in histopathological assessments were labeled as follow: thick red arrow—hair follicle, thick black arrow—sebaceous gland, thin blue arrow—neutrophil, blue head arrow—new epidermis, yellow arrow—dermis, thin white arrow—blood vessels, white star—collagen, GT—granulation tissue.

## Discussion

Currently, extensive scientific research is being conducted on a global scale for wound dressings. The scaffolds have only conductive properties with no impact on the induction of cellular proliferation, migration, and differentiation which are important components of the wound healing process. Accordingly, the need to use dressings that contain inductive substances (such as growth factors) is inevitable. At the beginning of wound formation, a high concentration of secreted PDGF growth factor could promote all phases of wound healing^21^.

The lack of growth factors affects cellular proliferation and differentiation. Growth factors are unstable and sensitive to proteases; as a result, they have a short half-life in the human body. The increase of growth factor stability during the wound healing process can be reached by: vesicles, 3D scaffolds, micro, and nanoparticle systems, and injection of recombinant human growth factors around the wound^22^.

The crystallographic structure of the PDGF-BB/PDGFR-β complex showed that three regions, including loops I and III of one monomer and loop II of the other monomer of PDGF-BB dimer, are involved in the interaction with the receptor^23^. Accordingly, several PDGF-derived peptides, which are mostly antagonists, have been approved and are designed to inhibit the PDGF signaling pathway^14,24^. Most of these peptides have either no disulfide bond or have only one disulfide bond. Disulfide bonding is one of the strongest covalent bonds that increase the stability of proteins and peptides^25^.

Structural studies of the PDGF-BB/PDGFR-ββ complex crystallography showed that there are two clamps at the end of the PDGF-BB, deriving from three inter-strand loops (L1 consisting of amino acid residues 25–38, L3 consisting of amino acid residues 78–81 and L2 consisting of amino acid residues 53–58). The carboxyl-terminal is involved in the interaction with the receptor. Since each clamp interacts with a PDGFR-β receptor molecule, both receptor monomers are involved in the interaction with the ligand. About 67% of the contact surface of the receptor/ligand is covered by the long arm (L1 and L3 loops), and the short arm of the clamp (the L1 loop and the C-terminal) covers the remaining contact area. Parts of the N-terminal of the ligand are also involved in establishing the contact surface with the receptor. The presence of hydrophobic interactions through amino acid residues Tyr205 and 207, Phe136 and 138 of PDGFR-β and Tyr40, Leu38, Ile75 and 77, and Pro82 of PDGF-B play a significant role in establishing the surface ligand/receptor contacts^26^. In the current project, two repeats of 18 amino acids (Gln71–Thr88) from the third loop of PDGF-B were considered for designing the fusion peptide. The sequence derived from PDGF-B has three parts: Gln71–Ile77, Pro82–Thr88 (the N-terminal and the C-terminal of Loop III, respectively, for the stability of the loop structure), and Val78–Lys81 (the functional site of Loop III).

We constructed the dimeric form of PDGF-BB-derived peptides from two beta sheets and a middle loop (loop III) like the real structure of dimeric PDGF-BB. The main reason for choosing this loop and the beta sheets on both sides is based on several studies. They have shown that this domain is the most important and functional domain for binding PDGF to the receptor. Since the PDGF is a homodimer, and according to crystallographic studies, the distance between the two loop III in the two monomers is about 75 Å. For this purpose, in this project, we tried to choose two similar domains and fused them using a large linker to mimic this distance. Our bioinformatics studies showed that the distance between two loops in the chimeric peptide is more than 55 Å. For the second Item, to design of chimeric peptide, we fused the two distinct PDGF derived with the hydroxyapatite (HA) affinity peptide as spacer for maintaining this suitable distance and two same peptides as tags in amino and carboxyl-terminal of chimeric form, that could be carried by HA scaffold component for future studies. This spacer and Two tags can increase the molecular size of fusion peptide for facilitating its expression as well as decrease wash out from the scaffold. The fusion peptide was expressed in the SHuffle^®^ *E*. *coli* strain, and its effect on the L929 cell line and in the animal model of wound healing was investigated. The goal of this study was to induce the PDGF signaling pathway with only its binding domain to reduce the size of the growth factor and subsequently increase its permeability and effectiveness. Four amino acids of cysteine were placed to form two disulfide bonds in the amine and carboxyl-terminal of the structure that provide proper stability by closing these regions. In addition, hydroxyapatite and His6x were also added to the construct.

The G_4_S linker was used for binding different parts of the fusion peptide. This flexible linker is superior to other non-flexible linkers because it does not have a negative effect on the proper folding of the structure and binding of the peptide to its receptor. The presence of small charged amino acids such as serine, threonine, and glycine are suitable for linkers. Small amino acids with polar charges create hydrogen bonds with aqueous solvents, thus improving solubility and preventing aggregation. On the other hand, these small amino acids are not negatively involved in secondary structure formation and the folding of proteins due to the lack of side chains in their structure^27^.

The fusion peptide has three hydroxyapatite-affinity sequence repeats (SVSVGNKPSPRP). Crystalline hydroxyapatite is the main component of teeth, and the fusion peptide could specifically bind to it in the cross-section of the human tooth. Hydroxyapatite and its derivatives are used in tissue engineering as support for stem cell differentiation to osteogenic lines. We used the hydroxyapatite affinity tag of HA-6-1 in the fusion peptide structure to bind the peptide to teeth in dental implants and induce tissue repair^28^. Radiesse^®^ (Bioform Inc, USA) is a sterile, latex-free, non-pyrogenic, semi-solid, cohesive subcutaneous, injectable implant whose main component is synthetic calcium hydroxyapatite, a biocompatible material with more than 20 years of use in medicine. This product has FDA approval and is used in the United States for beauty products^29^.

Although the molecular weight of the recombinant fusion peptide is 12.29 kDa, the fusion peptide band on the SDS-PAGE gel is about 17 kDa which is probably due to the presence of 10% lysine in its structure. Many positively charged amino acids cause a decrease in the total negative charge of the protein and eventually move slower than its actual weight on the SDS-PAGE gel^30^.

Due to the small structure of the His_6x_ sequence (less than one kDa), there is no change in the structure and function of purified protein. In vivo studies have also shown that the immunogenicity of the His_6x_ tag is very low. It is only possible to cover the epitope regions in the antibody fragments^31^. Many previous studies have used His_6x_-labeled proteins in animal studies, which indicates a lack of immunogenicity of this sequence in the structure of a variety of proteins and peptides^32,33^. TGFβ1^34^ and VEGF^35^ are among the growth factors purified by the His_6x_ tag and used directly for animal studies.

In previous studies, the effect of different concentrations (0.1–10 µg ml^−1^) of each of the growth factors, including PDGF^36^, epidermal growth factor (EGF)^37^, β-modifying growth factor^38^, and insulin-like growth factor (IGF)^39^ on increasing proliferation of different cell types has been shown. In this study, the treatment of the L929 fibroblast cells with the fusion peptide showed an increase in viability. Therefore, the fusion peptide in this study has a similar function to normal.

Growth factors, especially PDGF, cause fibroblast and epithelial cells to migrate in response to chemical factors (chemotaxis)^40^. Growth factors' effect, especially PDGF, on cell mobility has been investigated in previous studies. Most PDGFs^41^, EGFs^42^, β-modifying growth factor^43^ and FGF^44^ have increased cell mobility and migration.

Many previous studies have investigated the repairing ability of growth factor-derived human recombinant growth factors on wound healing in animal models. In most studies, wound regeneration was observed in animals after treatment with different doses of PDGF-BB locally, by injection, or by using expression vectors transferred to the wound site. In these studies, it was noted that healing and regeneration time was shortened after PDGF-BB treatment compared to the negative control group^45–48^. In previous studies, all PDGF structures (mostly BB type) were generated to investigate their effects on the wound. However, in this study, only the PDGF-BB binding region was examined.

This study also showed a decrease in the number of neutrophils at the wound site on days 7 and 12 due to the shift from the inflammatory phase to the repair and regeneration phase^49^. Skin regeneration was the result of epithelialization on days 7 and 12. Studies have shown that PDGF-BB has an effect on granulation enhancement during the first days of treatment^50^; It also stimulates epithelialization in the wounded tissue after the first week of treatment with PDGF-BB^51^.

Collagen III is the first protein synthesized in the early stages of wound healing that is replaced later on by collagen I, the skin's predominant collagen. Collagen production begins at the granulation stage with the migration of fibroblasts to the wound and continues until the final stage of wound healing. Changes in the collagen structure and the transformation into more complex structures are performed to restore the skin's tensile strength. Collagen regeneration continues for months after wound closure and if all processes are executed without any issues, the tensile strength of repaired tissue increases to about 80 to 85% of normal tissue^52,53^.

Our results on the effect of PDGF-BB-derived fusion peptide indicated its impact on angiogenesis. According to these results, the fusion peptide increased the growth and proliferation of endothelial cells due to the role of this peptide in the coaxial process; it caused the orientation of endothelial cells to shift primary tubular structures toward restored tissue at the site of the wounds^54^.

Our Histological studies showed an increase in tissue collagen^55,56^ and angiogenesis^57^ in the rat treated with recombinant PDGF-BB. Collagen I is known to strongly stimulate angiogenesis in vitro and in vivo through the involvement of specific integrin receptors. The pro-peptide C fragment of collagen I absorbs endothelial cells and significantly induces angiogenesis in wound healing^58^. As our results showed, the rate of angiogenesis in the treatment group increased significantly compared to the control group. Considering the similar increase in collagen production levels, it can be concluded that the increase in angiogenesis in the treatment groups on days 3, 7, and 12 is directly related to the increase in collagen levels.

There are limitations in this research, such as the total number of rats investigated and the lack of investigation with different peptide concentrations, which are recommended in other research.

## Conclusion

Our results indicated that PDGF-BB-derived fusion peptide could have a positive impact on wound healing. On the cellular level, our results showed an increase in the growth and migration of treated fibroblasts with the fusion peptide compared to the control group. The therapeutic effects of the designed fusion peptide on wound healing in rats showed that the peptide healed the wound, increased granulation tissue, and induced re-epithelialization, angiogenesis, and increased collagen production in rat models. In addition to the improvement of wound healing quality, our peptide also reduced the wound healing time in the treatment groups (especially on days 7 and 12) compared to the negative control groups.

### Supplementary Information

Supplementary Figures.

## Data Availability

All data analyzed during this project are included in this manuscript.
