# Supplementary material for: Functional characterization of the dimeric form of PDGF-derived fusion peptide fabricated based on theoretical arguments
Source: Sci Rep. 2024 Jan 10;14:1003. doi: 10.1038/s41598-024-51707-2 (PMC10781716; doi:10.1038/s41598-024-51707-2)
Supplement: Supplementary file 1 — Supplementary Figures. [file 41598_2024_51707_MOESM1_ESM.pdf]

Functional characterization of the dimeric form of PDGF-derived fusion peptide fabricated based on theoretical arguments

Maryam Sadeghi-Ardebili[1], Sadegh Hasannia[2*], Bahareh Dabirmanesh[2], Ramazan Ali Khavari-Nejad[1]

[1]Department of Biology, Science and Research Branch, Islamic Azad University Tehran, Iran
[2]Department of Biochemistry, Faculty of Biological Science, Tarbiat Modares University, Tehran, PO Box 14115-175, Iran

*Corresponding author: Sadegh Hasannia, hasannia@modares.ac.ir

Supplementary Figure3)

a

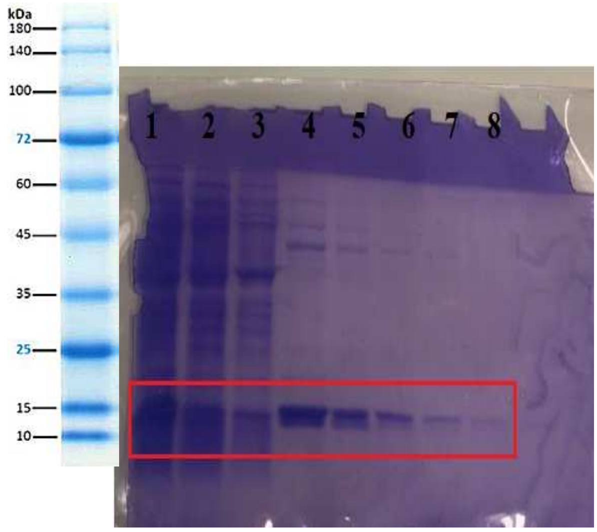

b

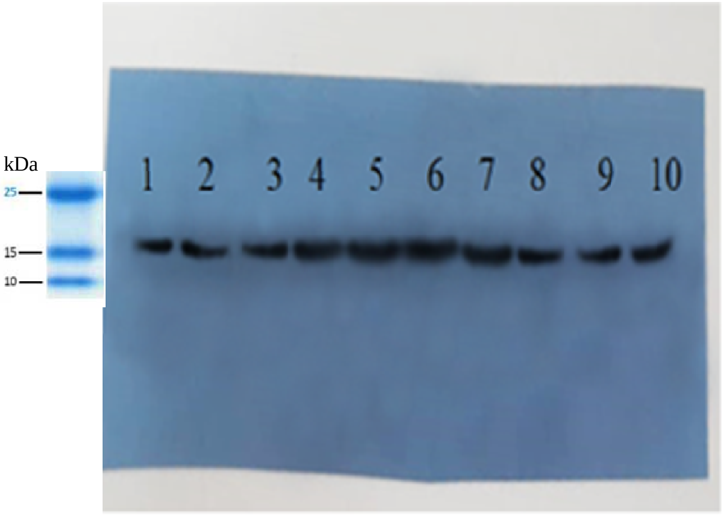

Figure 3) SDS-PAGE analysis of expression and purification of the fusion peptide by Ni-NTA agarose lane1: supernatant of *E. coli* lysate, lanes 2 and 3: washing buffer with 25 mM imidazole, lanes 4-8: elution buffer using 250 mM imidazole,
b) western blot analysis, lanes 1-4: soluble peptide, lanes 5-8: purified peptide, lanes 9 &10: native PDGF-BB as positive control.

Figure 3S1)

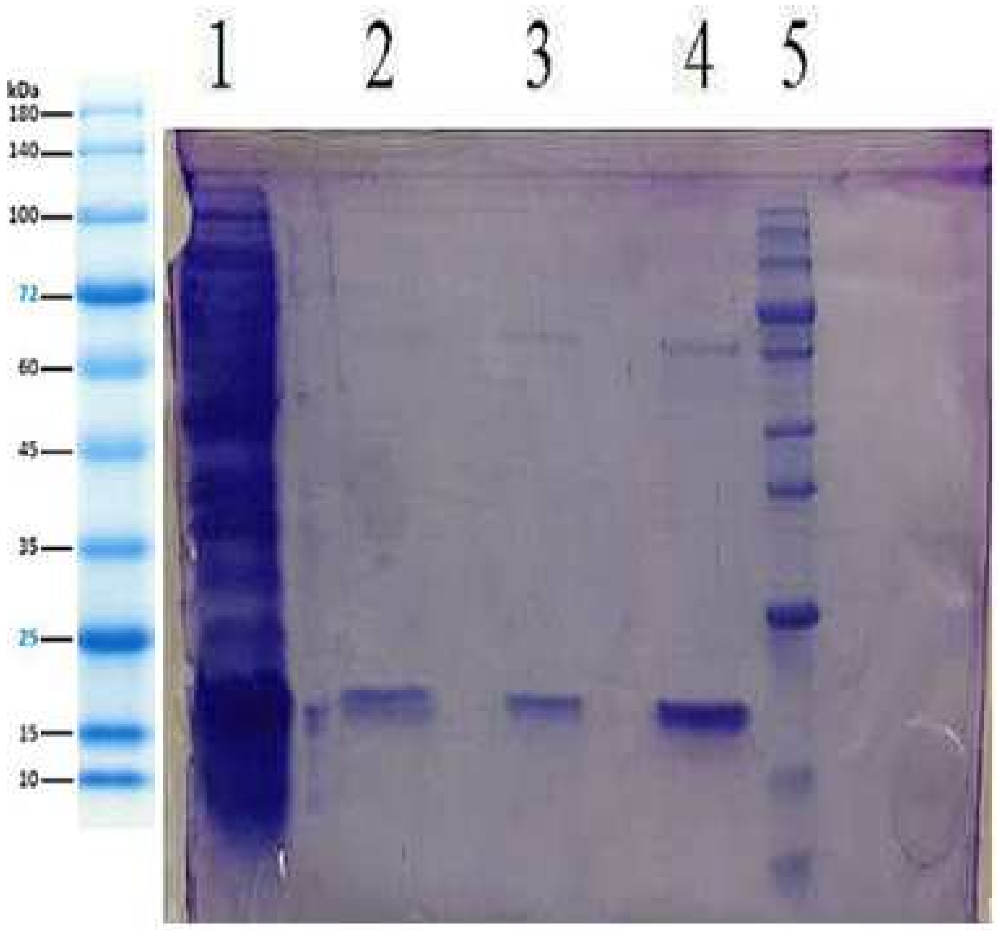

Figure 3-S1) SDS-PAGE analysis of expression and purification before and after dialysis and concentration.
Lane 1) peptide expression. lane2: purified peptide before dialysis. Lane 3: purified peptide after dialysis. lane 4: purified peptide after concentrating on PEG 6000. Lane 5: protein marker.

Figure 3S2)

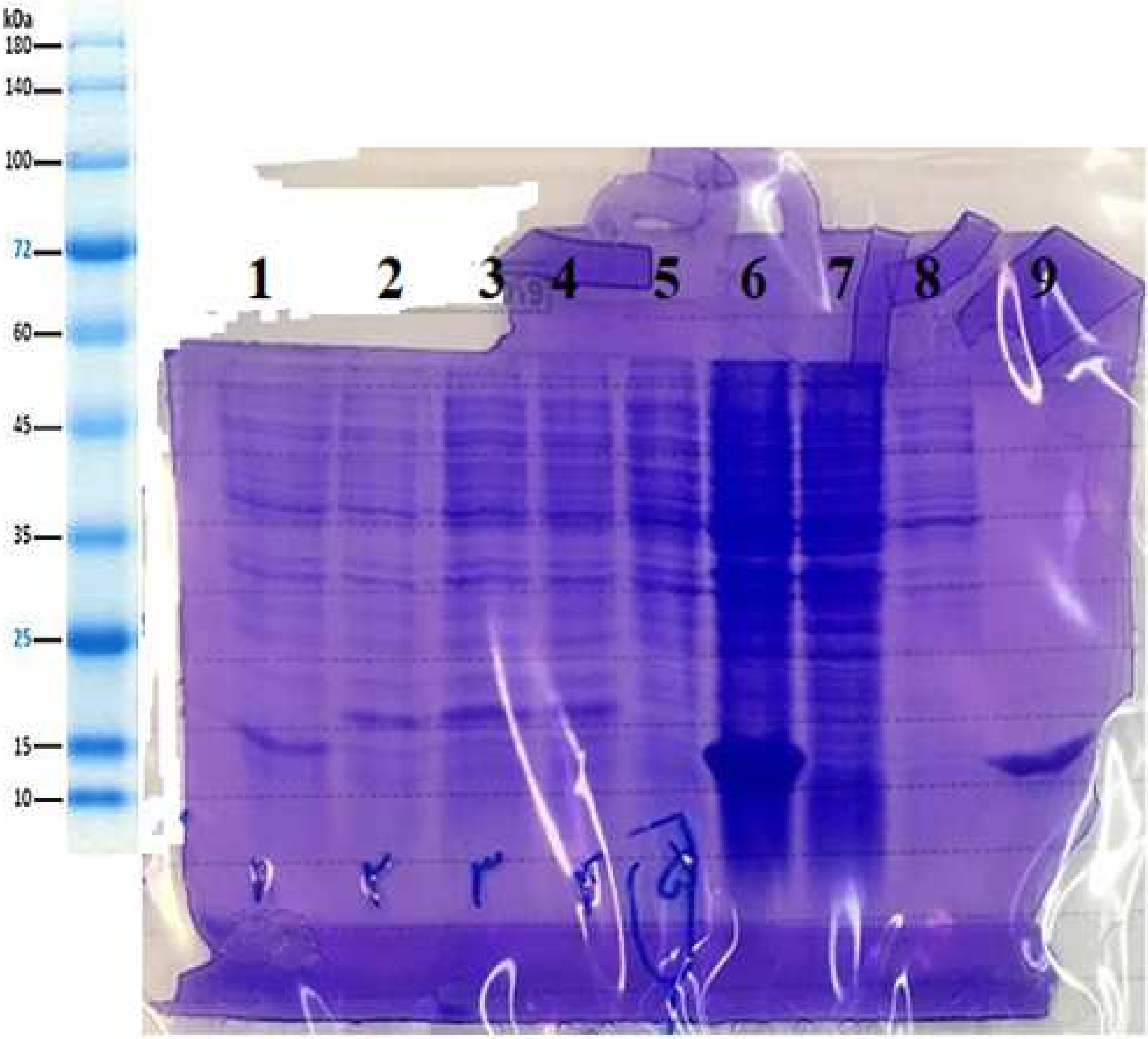

Figure 3-S2) SDS-PAGE analysis of expression and purification. Lanes 1-4: peptide expression. Lane5: negative control . lanes 6-9: related to other proteins to control the study procedure.

Figure 3S3)

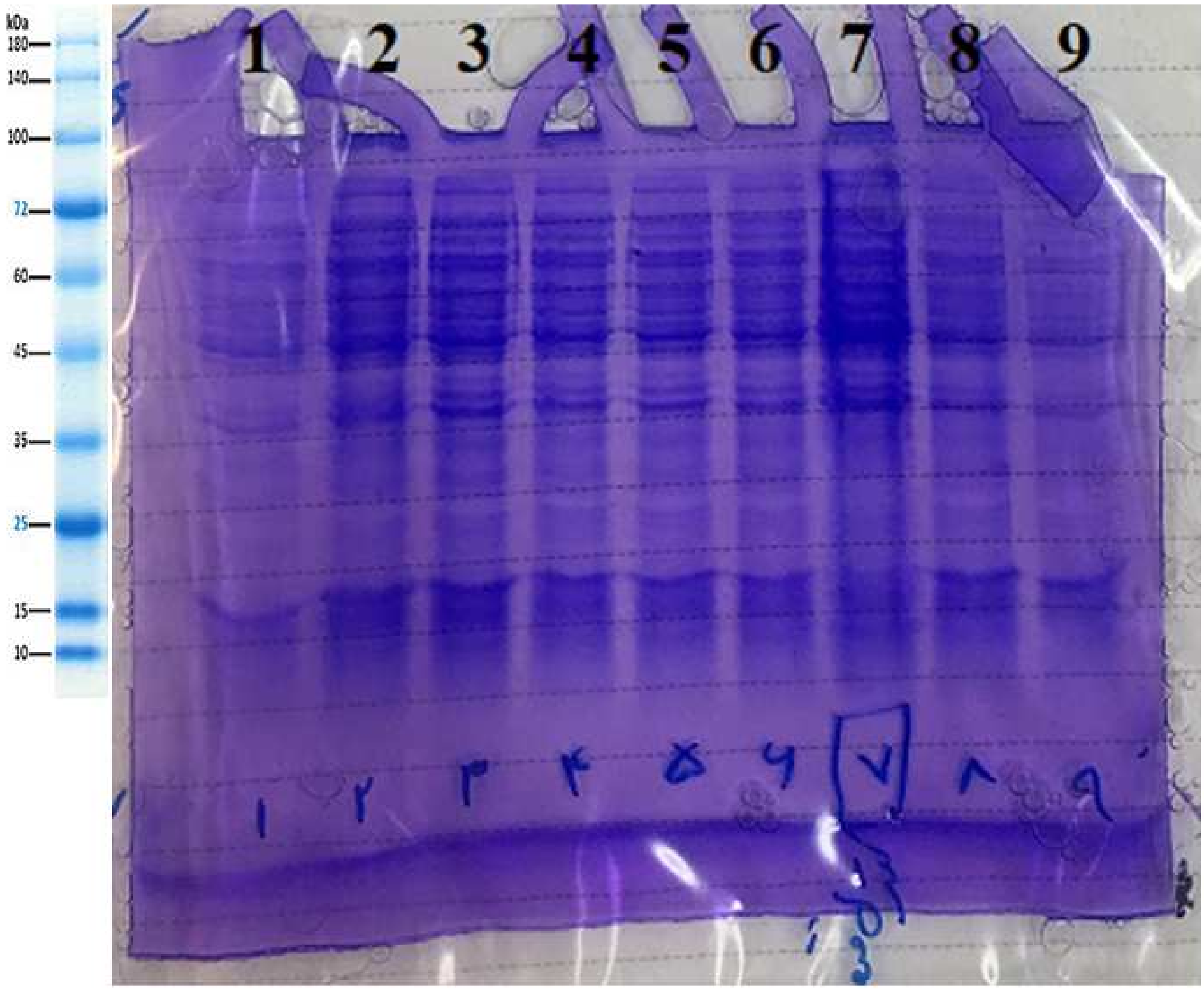

figure 3-S3) SDS-PAGE analysis of expression of fusion peptide is shown in lanes 1-6 and lanes 8-9. lane 7) negative control.

Figure 6)

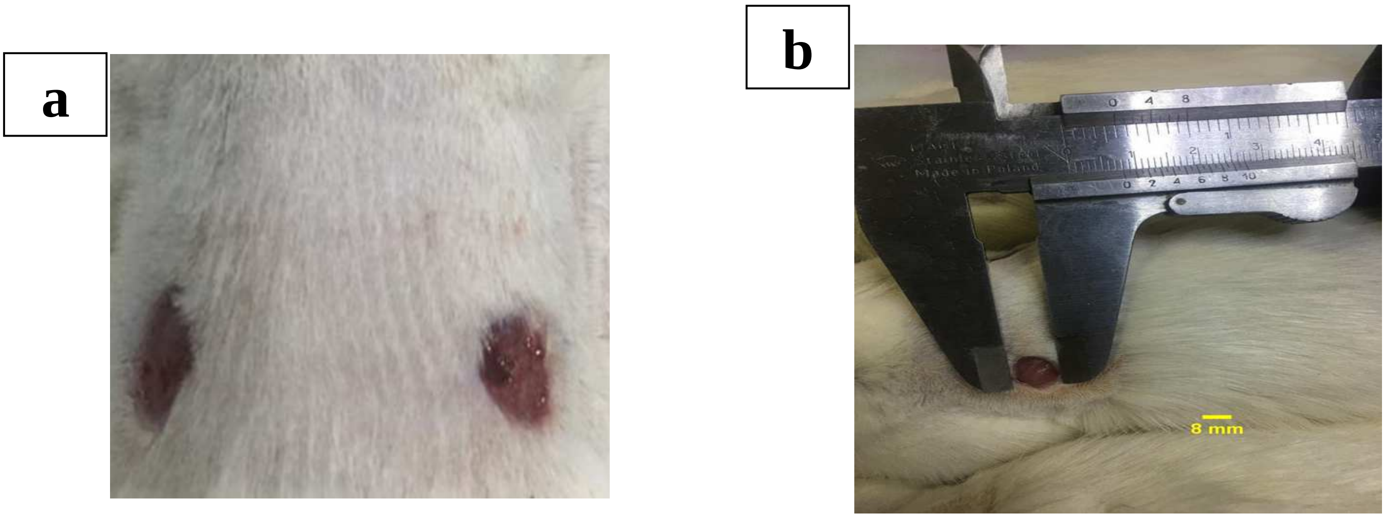

Figure 6. Macroscopic photo of a) day 0 rat model. Two full-thickness wounds (Left and Right) was created on the back of each rat model b) The clear caliper size of day 0.
